# Data and methods to calculate cut-off values for serum potassium and core temperature at hospital admission for extracorporeal rewarming of avalanche victims in cardiac arrest

**DOI:** 10.1016/j.dib.2019.104913

**Published:** 2019-12-02

**Authors:** Markus Falk, Hermann Brugger, Pierre Bouzat, Mathieu Pasquier, Peter Mair, Julia Fieler, Tomasz Darocha, Marc Blancher, Matthieu de Riedmatten, Peter Paal, Giacomo Strapazzon, Ken Zafren, Monika Brodmann Maeder

**Affiliations:** a Institute of Mountain Emergency Medicine, EURAC Research, Via Ipazia 2, 39100, Bolzano, Italy; b Medical University Innsbruck, Anichstraße 35, 6020 Innsbruck, Austria; c International Commission for Mountain Emergency Medicine ICAR MEDCOM, Italy; d Department of Anaesthesiology and Critical Care, Grenoble Alps Trauma Center, University Hospital of Grenoble-Alpes, 38043 Grenoble Cedex 09, France; e Emergency Service, Lausanne University Hospital Center, BH 09, CHUV, 1011 Lausanne, Switzerland; f Department of Anaesthesiology and Critical Care Medicine, Medical University Innsbruck, Anichstraße 35, 6020 Innsbruck, Austria; g Division of Surgical Medicine and Intensive Care, University Hospital of North Norway, 9037 Tromsø, Norway; h Anaesthesia and Critical Care Research Group, The Artic University of Norway, 9037 Tromsø, Norway; i Department of Anaesthesiology and Intensive Care, Medical University of Silesia, Medykow 14, 40-752 Katowice, Poland; j Department of Emergency Medicine, University Hospital of Grenoble-Alpes, 38043 Grenoble Cedex 09, France; k French Mountain Rescue Association ANMSM, 38043 Grenoble Cedex 09, France; l Department of Emergency Medicine, Sion Hospital, 1950 Sion, Switzerland; m Department of Anaesthesiology and Intensive Care, Hospitallers Brothers Hospital, Paracelsus Medical University, Kajetanerplatz 1, 5020 Salzburg, Austria; n Department of Emergency Medicine, Stanford University School of Medicine, Stanford, CA, USA; o Department of Emergency Medicine, Inselspital, Bern University Hospital, University of Bern, Freiburgstrasse 16C, 3010 Bern, Switzerland

**Keywords:** Avalanche, Accidental hypothermia, Core temperature, Cut-off value, Extracorporeal life support, Serum potassium, Triage

## Abstract

The data and estimation methods presented in this article are associated with the research article, “Cut-off values of serum potassium and core temperature at hospital admission for extracorporeal rewarming of avalanche victims in cardiac arrest: a retrospective multi-centre study” [1]. In this article we estimate recommended cut-off values for in-hospital triage with respect to extracorporeal rewarming. With only 6 survivors of 103 patients collected over a period of 20 years the ability to estimate reliable threshold values is limited. In addition, because the number of avalanche victims is also limited, a significantly larger dataset is unlikely to be obtained. We have therefore adapted two non-parametric estimation methods (bootstrapping and exact binomial distribution) to our specific needs and performed a simulations to confirm validity and reliability.

**Table:** Specifications Table

Subject area	Emergency medicine
More specific subject area	Avalanche accident, hospital triage
Type of data	Figures, method description
How data was acquired	Simulations
Data format	Raw and figure
Experimental factors	Fitted distribution functions to the observed parameter values
Experimental features	Monte-Carlo simulations with 1000 samples, each consisting of 100 simulated values, 6 for survivors and 94 for nonsurvivors.
Data source location	Bolzano, Italy, Eurac Research
Data accessibility	All data is included with this article.
Related research article	Brugger H, Bouzat P, Pasquier M, Mair P, Fieler J, Darocha T, Blancher M, De Riedmatten M, Falk M, Paal P, Strapazzon G, Zafren K, Brodmann Maeder M. Cut-off values of serum potassium and core temperature at hospital admission for extracorporeal rewarming of avalanche victims in cardiac arrest: a retrospective multi-centre study. Resuscitation. 2019 Jun; 139:222–229 [1].

**Table:** 

**Value of the Data** • This Data in Brief report provides simulated data and describes methods for estimating upper or limits when the number of events is small, but the overall sample size is sufficiently large. • We provide code that calculates cut-off values. • The proposed methods may be useful for researchers who need to set upper or lower limits when the number of events is low.

## 1 Data

**Fig. 1:**
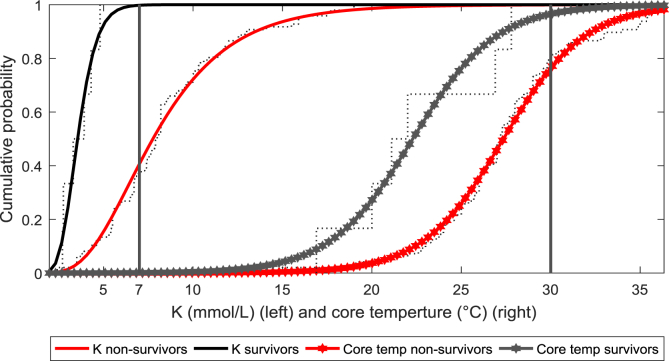
Fitted cumulative distribution functions for serum potassium level (K) and core temperature (Core temp). The best fit for potassium level was a log normal distribution (parameters for survivors: mean = 1.26, scale = 0.24 mmol/L, nonsurvivors mean = 2.04, scale = 0.45 mmol/L) and the logistic function for core temperature (parameters for survivors mean = 22.3, scale = 2.3 °C, nonsurvivors mean = 27.5, scale = 2.3 °C). The actual recommended cut-offs are marked by vertical lines.

**Fig. 2:**
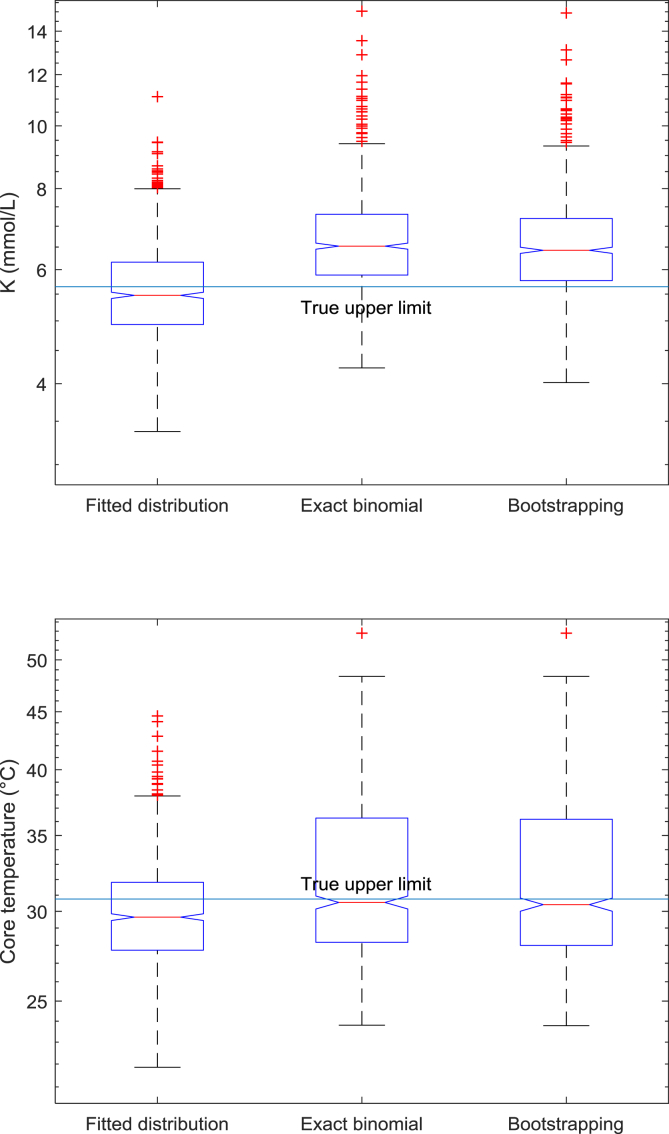
Monte Carlo simulations, 1000 simulation runs, for serum potassium (upper panel denoted as K) and core temperature (lower panel). Cut-offs from sampled survivor data correspond to the 97.5th percentile of the fitted underlying distribution function, while cut-offs from bootstrapping and binomial distribution use the observed maximum value in survivors to identify the corresponding percentile in the overall sampled data and uses the upper limit of the respective 95% CI as cut-off. The blue horizontal line shows the true cut-off (97.5th percentile of the distribution function used for simulation).

Fig. 1 shows the distribution functions of two variables of interest used for the simulations. Respective data is provided separately as a Matlab figure file “K_T_fit_DiB_fig1.fig”. Fig. 2 shows the outcome of Monte-Carlo simulations using the provided Matlab function “simcutoff.m”, which can be adapted to own data and for upper limit estimation we provide the Matlab function “getcutoff.m”.

## 2 Experimental design, materials, and methods

Our subjects were 6 survivors and 97 nonsurvivors. Our goal was to find cut-off values for serum potassium and core temperature that would differentiate between survivors and nonsurvivors. Although distribution functions could have been fitted to the data for a small number of events (survivors), the reliability would have been questionable and the resulting confidence interval (CI) for each measure would have been unreliable due to under- or overestimated variance. For each measure, the true standard deviation (SD) could be much smaller or much larger than the calculated value. For instance, in the case of 6 observations (n = 6), assuming a normal distribution, the 95% CI for the estimated SD runs from 0.62 x SD to 2.45 x SD. Thus, with an observed mean potassium of 3.6 ± 0.9 mmol/L, using 2.45 as a correction for SD, the respective 95% CI would range from −2.1 to 9.3 mmol/L, which is far beyond the range of possible values. Nonparametric methods and bootstrapping have the advantage that no assumptions about the underlying distribution function must be made, but reliability is still problematic.

The only information about a possible cut-off for survivors is the observed maximum value. Using all observations (survivors and nonsurvivors) a percentile can be obtained for the observed maximum value and a respective CI can be calculated. With this approach the missing information regarding variability in survivors is replaced by variability in the overall sample. However, the observed maximum value is itself unreliable. Therefore, a correction must be applied. To observe at least one value above the 80th percentile in six observations has a probability of 73% (binomial distribution, 80% is used as in the power assumption) and we used the reciprocal of this probability (1.4) as a correction factor. Instead of the observed percentile percentage we used the percentage multiplied by the correction factor and the upper limit of the obtained CI for the percentile as a conservative, but safe, estimate of the cut-off value.

The estimation steps for the cut-off value of a single parameter are as follows:

1) determine the maximum value (M) in survivors
2) obtain the percentage (P) of all observations in the overall sample less than or equal to the maximum value M
3) if P is greater than 50%, the maximal allowable over-triage rate, change P to 50%
4) multiply P by 1.4 and obtain a new percentage (P′)
5) calculate the 95% CI for P′ using bootstrapping (bias corrected percentile method) and optionally the exact binomial distribution method [2].
6) use the upper limit of this CI as the cut-off value.

### 2.1 Simulations

First, we fitted distribution functions to the observed serum potassium and core temperature values, separately for survivors and nonsurvivors (Fig. 1). The best fitting distribution function was a log normal distribution for serum potassium and a logistic distribution for core temperature. Using these functions, the 97.5th percentile can be calculated and served as the calculated true cut-off value. Furthermore, we used these functions to perform Monte-Carlo simulations with 1000 samples, each consisting of 6 random values for survivors (as observed in Ref. [1]) and 94 for nonsurvivors. For each sample we fitted the respective type of distribution function to simulated survivor data and used the respective 97.5th percentile as cut-off value. This corresponds to the conventional approach. Additionally, for each sample we used the maximum value in survivors to obtain the corresponding percentile in the overall sampled data, applied the correction factor and calculated the upper limit of the 95% CI using bootstrapping and the binomial distribution. As shown in Fig. 2, using survivor data alone, the conventional method will underestimate the true cut-off for serum potassium, while the proposed bootstrapping and binomial method is more conservative, as desired. For core temperature, we obtained similar results, but all methods underestimated the true cut-off. We therefore concluded that the proposed bootstrapping and binomial methods are safe for determining optimal cut-off values.

## Conflict of Interest

The authors declare that they have no known competing financial interests or personal relationships that could have appeared to influence the work reported in this paper.
